# Effects of Freeze–Thaw Pretreatment Combined with Hot Air on Snake Gourd (*Trichosanthes anguina* L.)

**DOI:** 10.3390/foods13131961

**Published:** 2024-06-21

**Authors:** Dandan Gu, Xiao Li, Mingyue Dong, Wenxuan Ji, Zihao Yan, Ting Zhao, Min Zhang, Peng Liu, Panpan Yue, Guanghua Mao, Liuqing Yang

**Affiliations:** 1 School of Chemistry and Chemical Engineering, Jiangsu University, 301 Xuefu Rd, Zhenjiang 212013, China; 2 School of the Environment and Safety Engineering, Jiangsu University, 301 Xuefu Rd, Zhenjiang 212013, China

**Keywords:** snake gourd, freeze-thaw, drying characteristics, active components, bioactivity capacities

## Abstract

Snake gourd is a seasonal vegetable with a high water content and medicinal value, but the short harvest period limits the large-scale application of snake gourd. Therefore, the effects of freeze–thaw pretreatment (FT) combined with hot air (HD) on the drying characteristics, active ingredients and bioactivities of snake gourd were investigated. The results showed that FT pretreatment reduced browning and shortened the drying time by 44%; the Page model was the best fit for describing the drying process. The polysaccharide contents (21.70% in alcoholic extract (TG1) and 44.34% in water extract (TG2)) and total phenol contents (1.81% in TG1 and 0.88% in TG2) of snake gourd pretreated by FT-HD were higher than those of snake gourd pretreated by the corresponding HD treatment. The FT pretreatment decreased the molecular weight of snake gourd polysaccharides and increased the molar ratio of glucose. The extracts pretreated by FT-HD showed greater chemical, cellular antioxidant capacity and α-amylase and α-glucosidase inhibition than those pretreated by HD. FT-HD can be recommended for achieving a short drying time and high quality of snake gourd and can be used for the drying of other fruits and vegetables.

## 1. Introduction

Snake gourd (*Trichosanthes anguina* L.) is an annual, monoecious, herbaceous vine crop belonging to the Cucurbitaceae family [1]. It contains a variety of biological compounds, including polysaccharides, flavonoids and polyphenols, with anti-oxidant properties and benefits including immune enhancement and the prevention and alleviation of diabetes, among other biological activities [2]. It is native to India [1], but now is cultivated all over the world. The snake gourd picking period is from June to October, and it is mainly consumed as fresh food. However, due to the short harvesting period, high water content and short shelf life of snake gourd, snake gourd resources are seriously wasted and its economic benefits and large-scale use are limited.

Drying is one of the most effective processing methods to extend the shelf life of vegetables [3,4]. HD has been widely used in fruit and vegetable drying due to its advantages of being low cost and a simple process and easy operation. Tang et al. [5] found that HD dried goji exhibited relatively high polysaccharide content and antioxidant activity. Meng et al. [6] found that the water-soluble polysaccharide content and antioxidant properties of HD dendrobium officinale were superior to those of the same plant pretreated by vacuum freeze drying. Whereas the waxy hydrophobic layer of fruits and vegetables during HD hinders the transport of water from the interior of the product to its surface and limits the heat transfer to the interior fruit portion, slowing down the drying rate and increasing the drying time, and prolonging the exposure to high temperatures, may lead to a significant decrease in the product quality [7]. It is reported that the polysaccharide obtained from *Agaricus blazei Murrill* via the vacuum freeze-drying method has stronger antioxidant abilities as compared with that obtained via HD and solar drying [8]. In order to improve the drying efficiency and quality of HD, drying pretreatment is widely used. Currently, several pretreatment methods have been applied to dry fruits and vegetables [9,10]. Conventional pre-treatments include hot water bleaching, hypertonic solutions, alkaline solutions, sulphate and acid solutions. Although these pre-treatments have a positive impact on the drying process by reducing drying time and improving quality, they can also lead to quality loss, poor rehydration, structural breakdown, nutrient loss and high energy consumption. In addition to this, the application of some pretreatment technologies still requires additional equipment such as ultrasonic generators, pulsed electric field equipment, etc., which increases the overall cost of the drying process. FT pretreatment is the process of freezing and re-dissolving food ingredients at low temperatures. The equipment requires only a refrigerator and does not increase the risk of contamination to the environment. FT pretreatment technology is easy to operate and has low equipment cost, which has good research application prospects. In the FT pretreatment process, the water in the fruit and vegetable cells is converted into ice crystals, resulting in the change of cell membrane permeability and the destruction of cell wall structure, effectively shortening the drying time and improving the drying characteristics [11]. It has been reported that the adoption of FT pretreatment improved the functional properties of dried onion powder and reduced the drying time [12]. Zhang et al. [13] demonstrated that FT pretreatment increased the drying rate and reduced the total energy consumption (from 4.77 to 3.73 kW h/kg) of lotus root, and resulted in a porous structure of the final product with a lower shrinkage (from 83.39 to 73.04%), a lower hardness (from 41.67 to 24.49 N) and a crispy texture (from 0.36 to 0.17 s). FT pretreatment of garlic [14] and okra [15] has been shown to reduce drying time by disrupting cellular integrity and forming micropores that facilitate moisture diffusion. This has also been found to save energy, reduce shrinkage and loosen the texture of the final product. FT pretreatments are effective options for reducing drying time and improving the quality of the final product [16]. However, to the best of our knowledge, little research has been conducted on snake gourd, and the effect of drying methods on the drying quality of snake gourd has rarely been reported. Therefore, research on drying methods of snake gourd is urgently needed in order to reduce the waste of resources and increase the utilization and economic benefits.

In this study, the increase in drying rate of snake gourd was analyzed by drying kinetics and microstructure. The active ingredient content, monosaccharide composition and molecular weight of dried snake gourd extracts were determined to further investigate the mechanism of action of antioxidant activity (i.e., chemical methods and cellular modelling) and enzyme inhibitory activity of snake gourd. These studies may help in finding suitable drying techniques for expanding the application of snake gourd.

## 2. Materials and Methods

### 2.1. Materials and Chemicals

Fresh snake gourd was purchased from Xishuangbanna Dai Autonomous Region (Xishuangbanna, China); it was identified as snake gourd (*Trichosanthes anguina* L.) of the genus Juniperus, family Cucurbitaceae by associate Professor Yanmin Zou (School of Pharmacy, Jiangsu University). Mature snake gourds with uniform shape, tender green color, caliber of 1–2 cm and length of 1–1.5 m were selected. Hep G2 cells were purchased from Shanghai Institute of Cell Biology (Shanghai, China). Fetal bovine serum (FBS) was purchased from Gibco (Grand Island, NE, USA). Dulbecco’s modified Eagle’s minimum essential medium (DMEM) was purchased from Hyclone (Logan, UT, USA). A penicillin–streptomycin mixture was obtained from Hefei Bomei Biotechnology Co., Ltd. (Hefei, China). Standard chemicals of gallic acid and glucose were purchased from Sinopharm Chemical Reagent Co., Ltd. (Shanghai, China). Mannose (Man), rhamnose (Rha), galactose (Gal), arabinose (Ara) and glucose (Glc) were purchased from Sigma Chemical Co. (Saint Louis, MO, USA). Glutathione (GSH), superoxide dismutase (SOD), catalase (CAT), lactate dehydrogenase (LDH), reactive oxygen species (ROS) and malondialdehyde (MDA) assay kits were obtained from Nanjing Jiancheng Bioengineering Institute (Nanjing, China). All other chemicals and solvents were analytical grade.

### 2.2. Drying Experiments

#### 2.2.1. Freeze-Thaw Cycles Pretreatment

Snake gourds were evenly cut into 5 mm discs and placed in an ultra-low temperature refrigerator (DW-86L388J, Qingdao Haier Co., Ltd., Qingdao, China) at −20, −40, and −80 °C for freezing, and the temperature in the center of the samples was recorded every 1 min using a digital thermometer (TA612C, Suzhou TASI Electronics Co., Ltd., Suzhou, China) until the sample’s internal temperature became constant and freezing was stopped. The samples were thawed immediately at 30 ± 1 °C until the internal temperature was constant. The process of freezing followed by thawing was one freeze-thaw pretreatment (FT1); this was repeated to obtain samples frozen and thawed 2 and 3 times (FT2, FT3). FT pretreatment methods are shown in Table 1.

#### 2.2.2. Hot Air Drying

**Figure 1:**
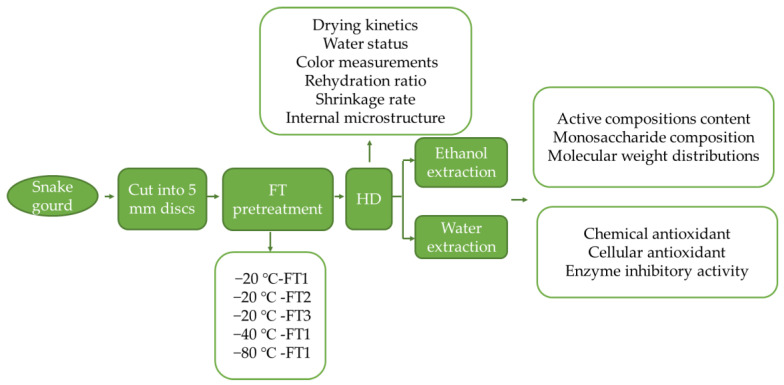
Drying protocol of snake gourd.

The sample was laid tightly flat on the partition in a constant temperature oven (Yiheng Experimental Instrument Co., Ltd., Shanghai, China) at a controlled temperature of 60 °C and the air speed was set to 1 m/s. During the drying process, the samples were weighed every 0.5 h until the moisture content reached below 8% to determine the total drying time and obtain dried samples. Three replicate drying experiments were conducted. The snake gourd drying protocol is shown in Figure 1.

### 2.3. Drying Characteristics Analysis

#### 2.3.1. Drying Kinetics

##### Determination of Moisture Ratio

The moisture content (MC) and moisture ratio (MR) of snake gourd during drying were calculated using Equations (1) and (2):

(1) $$MC=\frac{m_{t}-m_{d}}{m_{d}}$$

(2) $$MR=\frac{M_{t}-M_{e}}{M_{0}-M_{e}}$$

where, m_t_ and m_d_ are the mass of the sample at time t and equilibrium (g), respectively; M_0_, M_e_ and M_t_ distribution are the dry basis moisture content of the sample at initial time, equilibrium and t (g/g).

##### Establishment of the Mathematical Model of Drying Kinetics

The resistance to moisture migration is assumed to be uniformly distributed throughout the interior of the homogeneous isotropic fermented food material. Therefore, the diffusion coefficient D is independent of the water content. The corresponding volume contraction is assumed to be negligible and then Fick’s second law can be derived from the following equation:

(3) $$\frac{\partial M}{\partial t}=D\frac{\partial^{2}M}{\partial t^{2}}$$

The two-term exponential model is the first two terms of the general order solution of Equation (4). The Henderson and Pabis model is also known as the two-parameter exponential model. The model is a generalized cascade solution of Fick’s second law. The Lewis model is a Henderson and Pabis variant with a constant intercept. To compensate for its shortcomings, the Page and Logarithmic model is an experimental adaptation of the Lewis model. The Wang and Singh model is derived by simplifying the general grade solution of Fick’s second law and is a second order polynomial model. These have been successfully applied to explain the drying process of certain agricultural products.

The drying models (Table 2) was used to fit the variation of moisture ratio (MR) with time (t).

The parameters, i.e., coefficient of determination (R^2^), reduced Chi square (χ^2^), and root mean square error (RMSE), were applied to evaluate the fitting quality of different models obtained under different FT pretreatment conditions.

(4) $$R^{2}=1-\frac{\sum_{i=0}^{N}{\left(M_{exp,i}-M_{pre,i}\right)}^{2}}{\sum_{i=0}^{N}{\left(M_{exp,i}-{\bar{M}}_{pre,i}\right)}^{2}}$$

(5) ERMS=1N∑i=1NMexp,i−Mpre,i212

(6) $$\chi^{2}=\left.\frac{\sum_{i=1}^{N}{\left(M_{exp,i}-M_{pre,i}\right)}^{2}}{N-Z}\right.$$

where, M_pre,i_, $\bar{M}$_pre,i_, M_exp,i_, N and Z are predicted moisture ratio, predicted average moisture ratio, experimental moisture ratio, number of experimental data and the number of constants in the drying model, respectively.

#### 2.3.2. Water Status

The water status from transverse sections of the samples after FT pretreatment was determined by low-field pulsed nuclear magnetic resonance analyzer (LF-NMR) (NMI20-060V-I, Suzhou Nimag Analytical Instruments Ltd., Suzhou, China). A slice of snake melon was placed into a 60 mm glass tube and inserted into the NMR probe. The spin–spin relaxation time (T2) was measured using the Carr-Purcell-Meiboom-Gill (CPMG) sequence. The typical parameters were as follows: SF = 21 MHz, O1 = 134,555, P1 = 7.52 μs, SW = 200 kHz, RFD = 0.002 ms, TD = 598,404, PRG = 2, RG1 = 20, TW = 3000 ms, NECH = 17,000, P2 = 14.48 μs, TE = 0.8 ms and NS = 8.

#### 2.3.3. Color Measurements

The color profile was obtained using a color analyzer (ColorQuest XE, HunterLab, Reston, VA, USA). The light source was a pulsed xenon lamp. The d/8° geometry was used for reflection and transmission measurements. The color properties were quantified using characteristics denoted as L* (darkness to lightness spectrum), a* (redness to greenness spectrum), b* (yellowness to blueness spectrum) and ΔE (total color change) values. ΔE was calculated using the following formula:

(7) $$\Delta E=\sqrt{(L^{*}-{L_{0}^{*})}^{2}+(a^{*}-{a_{0}^{*})}^{2}+(b^{*}-b_{0}^{*}{)}^{2}}$$

where, $L_{0}^{*}$, $a_{0}^{*}\;$ and $b_{0}^{*}$ are the values of HD snake slices, while L*, a* and b* represent the values of snake slices after pretreatment drying with FT.

#### 2.3.4. Rehydration Ratio

The rehydration ratio of dried snake gourd slices was performed according to the method of Zhang et al. [13]. Samples (Dried Snake gourd slices) of 1 g (W_2_) were soaked in distilled water at 25 °C, and the weight of the samples were estimated at intervals of 30 min until the weight was stable (W_1_). RR was calculated according to the following equation.

(8) $$RR=\frac{W_{1}}{W_{2}}\times 100\%$$

where, W_1_ and W_2_ are the mass values (g) of the rehydrated and dried samples, respectively.

#### 2.3.5. Shrinkage Rate

The shrinkage rate of dried snake gourd powder was evaluated according to a previous study [13]. Briefly, dried quartz sand (60 mesh to 80 mesh) was placed in a graduated cylinder and compacted to a volume of 100 mL, then dried snake gourd sample (10.0 g) and above quartz sand (100 mL) were mixed and placed in a graduated cylinder, compacted and the volume increments measured.

(9) $$SR=\frac{V_{1}}{V_{0}}\times 100\%$$

where V_0_ and V_1_ represent the volume of the fresh and dried sample, respectively. The mass of fresh snake gourd was calculated by water content, and its volume V_0_ was calculated by adding a certain volume of distilled water to detect the volume increment.

#### 2.3.6. Internal Microstructure

Snake gourd powder was pasted on the conductive adhesive and sprayed with gold. The samples were observed using a field emission scanning electron microscope (JSM-7001F, Hitachi, Japan) amplified 350× under high vacuum conditions. The electron beam accelerating voltage of 2.00 kV.

### 2.4. Structure Characterization of Ethanol (TG1) and Water (TG2) Extraction

#### 2.4.1. Preparation of TG1 and TG2

TG1 and TG2 were prepared according to the report [23] with a minor modification. First, 10 g of snake gourd powder was extracted twice with ethanol (1:15, *w*/*v*) at 75 °C for 1 h. After centrifugating (6000 rpm, 15 min), the supernatant was collected, condensed and lyophilized to give TG1. Subsequently, the residue was extracted twice with boiling water (1:30, *w*/*v*), and the supernatant was collected, concentrated and precipitated with four times volume of cold anhydrous ethanol. The precipitate was obtained by centrifuging at 8000 rpm for 10 min) then being dissolved in distilled water and dynamically dialyzed in a dialysis bag (3500 Da) for three days. Finally, the solution in the dialysis bag was freeze-dried to harvest TG2.

#### 2.4.2. Determination of Active Compositions (Polysaccharide and Total Phenolic) Content

Polysaccharide content was determined by the phenol-sulfuric acid method, taking glucose as the standard [24]. First, 0.3 mL sample solution of snake gourd was added into 0.6 mL 5% phenol solution, and then 3.0 mL H_2_SO_4_ was added into the mixture. After the mixture was thoroughly mixed, the absorbance was measured at 490 nm after being placed in a water bath at 30 °C for 30 min.

Total phenolic content was evaluated by Folin–Ciocalteu colorimetric method using gallic acid as the standard [25]. First, 1.0 mL 0.1 moL/L folin phenol reagent was thoroughly mixed with 1 mL sample solution for 5 min, then 1.5 mL 7.5% Na_2_CO_3_ solution was added. After mixing, color reaction was performed for 2 h. Absorbance was measured at 765 nm.

#### 2.4.3. Determination of Monosaccharide Composition

Hydrolysis and acetylation of TG1 and TG2 was conducted using the method described by Wang et al. [26]. The product was analyzed by GC-MS (Aglient 6890N/5759B, Agilent Technologies, Santa Clara, CA, USA), and the applied temperature program was selected as follows: initial temperature 130 °C held for 5 min, then ascended to 240 °C at a rate of 4 °C/min and, finally, held for 5 min at 240 °C.

#### 2.4.4. Determination of Molecular Weight Distributions

The molecular weight distributions of TG1 and TG2 were determined by high-performance size exclusion chromatography (HPSEC) coupled to a multi-angle laser light scattering (MALLS, DAWN HELLOS II λ = 658 nm; Wyatt Technologies Corporation, Goleta, CA, USA). The HPSEC-MALLS was performed on an Agilent 1260 HPLC system (Agilent, Santa Clara, CA, USA) equipped with an Agilent G1362A refractive index detector (RID, Agilent) and two SEC columns (OHpak SB-806 M HQ and SB-805 HQ, 8 mm Φ × 30 cm, Shodex, Tokyo, Japan) in series at 25 °C. 0.1 M NaCl contain 0.02% NaN_3_ solution was used for elution at a flow rate of 0.5 mL/min. The solvents were filtered using 0.22 μm polytetrafluoroethylene membranes (Millipore, Burlington, MA, USA) prior to injection. The injection volume was 200 μL, and the refractive index increment was 0.138 mL/g.

### 2.5. Biological Activities of Dried Snake Gourd Extraction

#### 2.5.1. Chemical Antioxidant Evaluation

DPPH· and ABTS+ scavenging capacities were assessed using the method previously reported by Wang et al. [26].

First, 200 μL of DPPH-ethanol solution (0.1 mM) was added to 100 μL of TG1 and TG2 solution at different concentrations (0–1000 μg/mL). The mixture was kept at 37 °C for 30 min. The absorbance of the test mixture was tested at 517 nm; ethanol was used as a blank control instead of the sample solution.

(10) $$Scavenging\;rate\left(\%\right)=\frac{\left(A_{control}-A_{sample}\right)}{A_{control}}\times 100$$

Potassium persulfate (2.45 mM) and ABTS (7 mM) were mixed and incubated at 25 °C for 16 h. The mixture solution was diluted with deionized water until the absorbance at 734 nm was 0.70 ± 0.02. 50 μL of TG1 and TG2 solution (0–1000 μg/mL) was added to 100 μL of ABTS solution. The absorbance at 734 nm was measured after 6 min of reaction at 25 °C. Deionized water was used in place of the sample as a blank control. The ABTS scavenging effect was calculated as:

(11) $$Scavenging\;rate\left(\%\right)=\frac{\left(A_{control}-A_{sample}\right)}{A_{control}}\times 100$$

#### 2.5.2. Cellular Antioxidants Determination

##### Measurements of ROS Level

100 μL of Hep G2 cells at a density of 6 × 10^4^ cells/mL were added to a 96-well plate. The cells were incubated at 37 °C for 24 h, and then treated with 100 μL of different concentrations of TG1 or TG2 solutions, while the cells in the blank and model groups were treated with equal volumes of medium. After 24 h incubation, 100 μL of new medium containing 250 µmol/L H_2_O_2_ was added to each well, except for the blank group, and incubated for another 1 h. Then, 100 μL of DCFH-DA (10 μM) was added incubated at 37 °C for 30 min after the supernatant was removed. The fluorescence intensity was measured using a microplate reader (SYNERGY H4, BioTek, Winooski, VT, USA) with emission and excitation wavelengths of 520 nm and 485 nm, respectively.

##### Measurements of LDH, SOD, CAT, GSH and MDA

Hep G2 cells (6.0 × 10^5^ cells/well, 1mL/well) were cultured in 6-well plates under the same conditions as pervious section. The supernatant was collected for LDH assay, and the cells were harvested for protein extraction. The levels of SOD, CAT, GSH and MDA in cell protein extractions were determined by a commercial assay kit according to the kit manufacturer’s instructions.

#### 2.5.3. Inhibitory Activity against α-Amylase and α-Glucosidase Assay of TG1 and TG2

The α-amylase and α-glucosidase inhibitory activities were performed according to previous reported method [27]. For the assay of α-amylase inhibitory activity, 20 μL of TG1 or TG2 was mixed with 20 μL of α-amylase (0.1 mg/mL). The mixtures were incubated at 37 °C for 10 min. Then 40 μL of 0.1% starch (*w*/*v*) was added for 20 min at 37 °C. Afterward, 80 μL of 0.4 M HCl and 100 μL of 5 mM iodine (dissolved in 5 mM KI) was added. Absorbance was measured at 630 nm. α-amylase inhibitory activity (%) was estimated by the following formula:

(12) $$Inhibitory\;activity\left(\%\right)=1-\frac{A_{s}-A_{d}}{A_{c}}\times 100$$

where, A_s_ is the absorbance value of the mixture of extract, enzyme and starch, A_d_ is the absorbance of the buffer solution replacing the enzyme solution and A_c_ is the absorbance value of the buffer solution replacing the sample solution.

For the determination of α-glucosidase inhibitory activity, 50 μL of TG1 or TG2 was mixed with 50 μL of α-glucosidase solution (0.3 U mL^−1^) held at 37 °C for 20 min before adding and 50 μL of 5 mM α-p NPG solution, and the mixtures were incubated for 20 min at 37 °C in the dark. Afterward, 80 μL Na_2_CO_3_ was added to record the absorbance at 405 nm. α-glycosidase inhibitory activity (%) was calculated by the following formula:

(13) $$Inhibitory\;activity(\%)=1-\frac{A_{s}-A_{d}}{A_{c}}\times 100$$

where, A_s_ is the absorbance value of the mixture of extract, enzyme and PNPG, A_d_ is the absorbance value of the mixture after the buffer solution replaces the enzyme solution and A_c_ is the absorbance value of the buffer solution instead of the sample solution.

### 2.6. Statistical Analysis

Data were expressed as mean ± SD. The significance of differences was assessed using the Tukey test in one-way ANOVA using SPSS 15.0 statistical software. Statistical significance was defined as *p* < 0.05.

## 3. Results and Discussion

### 3.1. Effect of FT Pretreatment on the Drying Process

**Figure 2:**
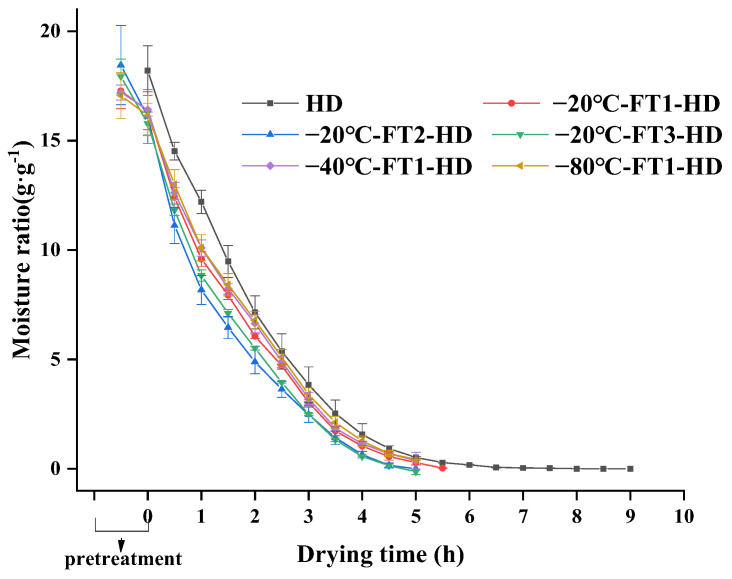
Curves of water content of snake gourd with HD and FT pretreatments.

The moisture ratio is an important parameter in the drying process, which reflects the rate change during the drying process. Figure 2 shows the moisture content profile of snake gourd slices under different pretreatment conditions. Compared with the HD, FT pretreatment greatly shortened the drying time and increased the drying rate of snake gourd slices. The conditions of −20 °C-FT2-HD showed the shortest drying time, which was reduced by 44% as compared with that in the HD. This is attributed to the fact that FT pretreatment can increase the drying rate by disrupting cell walls and by osmotic pressure. The higher freezing temperatures (−20 °C) have lower freezing kinetics, thus creating larger ice crystals [28]. The drying rate of −20℃-FT2-HD was greater than that of −20 °C-FT1-HD, which indicates that the drying rate accelerated with the increase in the number of freezing and thawing cycles; when the number of freezing and thawing cycles is three and the two have similar rates, the increase in drying rate is smaller. The results are in agreement with those of Feng et al. [14], which indicated that an increase in the number of FT pretreatments resulted in a faster drying rate for garlic.

The statistical parameters R^2^, χ^2^ and RMSE of the six drying models were compared. The values of the statistical analyses are summarized in Table 3. The R^2^ values of all the models are above 0.95 except for −80 °C-FT1-HD. The Page model was the optimal for drying, with R^2^ values greater than 0.99425, χ^2^ (1.57 × 10^−4^~6.73 × 10^−4^) and RMSE (0.0022~0.00741) values lower than those of the other models. Therefore, it was chosen to represent the thin layer drying characteristics of snake gourd. The Page was best fitted in FT pretreatment in grape HD, with the highest R^2^ as well as the lowest RMSE and χ^2^ [29]. Li et al. [30] found that the Page model was able to predict the water loss process of *gastrodia elata* tablets.

**Figure 3:**
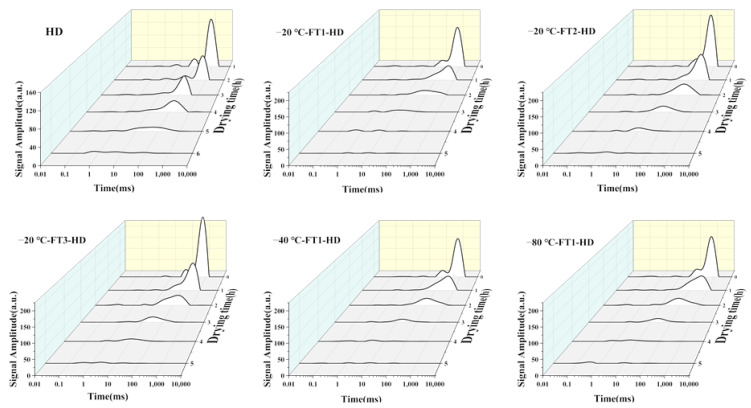
T2 relaxation spectra of snake gourd slices with HD and FT pretreatments. Yellow represents the variation of signal intensity with relaxation time, green represents the variation of signal intensity with drying time, and grey represents the variation of relaxation time with drying time.

LF-NMR can reveal water distribution in plant tissues by analyzing the transverse relaxation time T2. There are generally three types of water states in fruits and vegetables. The T2 relaxation spectra of 0.01–10 ms are for bound water, 10–100 ms are for immobilized water and 100–1000 ms are for free water. As shown in Figure 3, The FT + HD sample curves were shifted towards the left side of the relaxation time axis compared with HD. This indicates that FT pretreatment shortens the relaxation time of T2 samples, which is typical of water loss [7]. The T2 relaxation time was shortened in FT2 and FT3 compared with FT1. This is because the increased number of freeze-thaw cycles results in a weaker ability to bind to water molecules, giving them more degrees of freedom. The analysis of water distribution in thawed based on their T2 relaxation spectra is summarized in Table 4; FT + HD significantly increase the percentage of free water (from 81.77% to 90.21%) (*p* < 0.05). Among the pretreatment conditions, −20 °C-FT2-HD had the highest percentage of free water, whereas no significant difference was observed in comparison with other pretreatment conditions (*p* > 0.05). FT pretreatment accelerated the conversion of strongly bound water and immobilized water to free water. The increase in free water molecules may be related to changes in the structure and conformation of biopolymers within the cell. FT pretreatment leads to weaker binding to water molecules, giving them more degrees of freedom [7]. Guo et al. [7], in their study of FT pretreatment combined with HD dried garlic, found that the proportion of bound water decreased and the proportion of free water increased, suggesting that FT promoted the conversion of bound and fixed water to free water. In addition, Feng et al. [14] found that the T2 relaxation time of garlic was shortened and the water content of garlic slices was gradually reduced with the increase in the number of freeze–thaw cycles.

Color is a key factor in consumer acceptance. L* value represent the whiteness of the sample, and the L* values of FT + HD samples were significantly higher than HD samples, except for the −20 °C-FT1-HD and −20 °C-FT2-HD treatments. With the decrease in FT temperature and the increase in the number of FT cycles, L shows an increasing trend. a* value represents the red–green spectrum. The smaller the a* value, the greener the sample. It can be seen that in the dried product, the a* of FT + HD is less than HD, but there is no significant difference (*p* > 0.05). The b* value indicates a blue-yellow spectrum; the larger the b* value, the yellower the sample. The b* values of the FT + HD samples were significantly lower than those of the fresh product, except for the −80 °C-FT1-HD. The increase in a* and b* values in snake gourd may be attributed to the destruction of cellular structure and promotion of pigment entry into the tissue due to FT pretreatment [7]. The decrease in L* values and increase in b* values may be associated with enzymatic and non-enzymatic browning of the snake gourd during FT [31]. The FT pretreatments reduced browning to varying degrees, which may also be related to the reduction of drying time [32].

Dehydration causes changes in shrinkage and rehydration, which affects texture quality and consumer acceptance of the product. As can be seen in Table 5, The change curve of the rehydration ratio is shown in Figure S1 of the Supplementary Materials. FT + HD pretreatment significantly increased the shrinkage and decreased the rehydration rate of snake gourd (*p* < 0.05). With the increase in the number of freezing–thawing cycles, the shrinkage of the samples first increases and then decreases and the rehydration rate decreases. This suggests that a lower number of freeze–thaw cycles is beneficial in maintaining the shape of snake gourd. This is related to the damage of microstructure caused by FT pretreatment. After FT3 treatment, most of the cellular structure may be damaged, the force to support the tissue structure decreases rapidly, the tissue shrinkage increases and the rehydration rate decreases. The rehydration rate was higher at higher temperatures during freezing, but no significant difference in shrinkage was observed (*p* > 0.05). This may be caused by the ice crystals produced during freeze-thaw puncturing the cellular structure. The higher the temperature, the longer the ice crystals formed and the lower the rehydration rate of the slices [31]. This is in agreement with the results of Feng et al. [14]; FT pretreatment decreased the rehydration rate and increased the shrinkage of goji berries, and FT3 had the lowest rehydration rate and the highest shrinkage.

**Figure 4:**
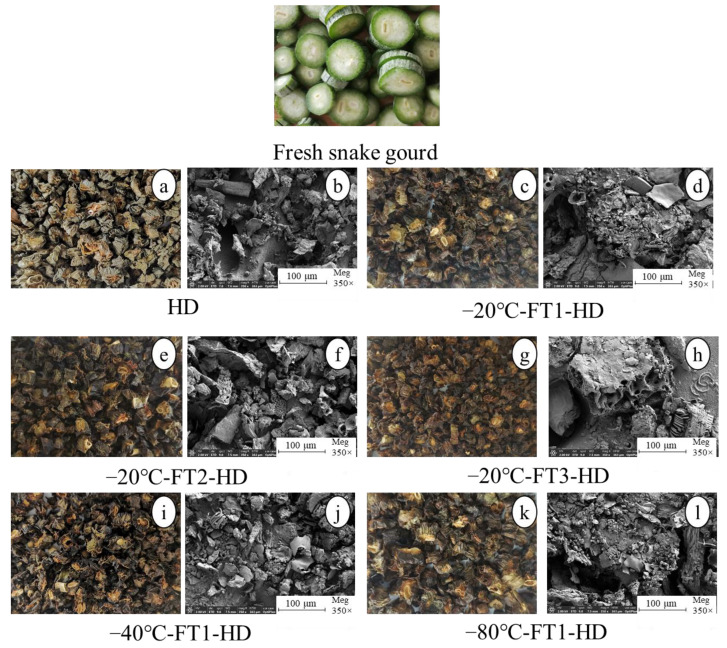
Digital photos and scanning electron micrographs of snake gourd slices with HD and FT pretreatments. (**a**,**c**,**e**,**g**,**i**,**k**): sample images; (**b**,**d**,**f**,**h**,**j**,**l**): scanning electron microscope images.

The microstructure of vegetables after drying can provide a reference for their mass transfer processes and mass changes [33]. It can be seen that the FT + HD samples showed tiny pores compared with the HD samples (Figure 4), and the lower the freezing temperatures, the faster rate of freezing and the smaller and fewer the holes. The higher the freezing temperature of the FT pretreatment, the longer it takes to freeze completely, the larger the ice crystals that form, the more damage to the tissue cells and the more porous they become. This is because the more FT circulates, the more ice crystals there are and the number of pores has increased [34]. This accounts for the accelerated drying rates of FT2 and FT3. The porous structure facilitates the rapid escape of moisture. Similar results were observed with FT pretreatment for garlic and lotus root, with disruption of the tissue structure and the appearance of micropores within [14,34].

In view of the faster drying rate of −20 °C-FT-HD, this pretreatment was subsequently used in a comparative study with HD.

### 3.2. Characterization of TG1 and TG2

#### 3.2.1. Active Compositions Content Analysis

Polysaccharide and phenolic compounds are natural antioxidants found in plants [34]. The polysaccharide and total phenolic contents of TG1 and TG2 are shown in Table 6. Polysaccharides have a greater affinity for water molecules due to the large number of polar groups in their molecules. Thus, TG2 contains high polysaccharide content. Phenols are soluble in warm water and ethanol, but they are thermally unstable. Therefore, TG1 shows a higher total phenol content. The polysaccharide and total phenolic content increased to varying degrees after FT pretreatment, significantly (*p* > 0.05). The −20 °C-FT2-HD sample ranked the highest in terms of polysaccharide and total phenol content. This indicates that FT pretreatment can improve the drying stability and extraction rate of the active components. The results were similar to those of Feng et al. [14]; for the lyophilized goji berries, the order of polysaccharides and total phenol contents was FT > control.

#### 3.2.2. Monosaccharide Composition Analysis

**Figure 5:**
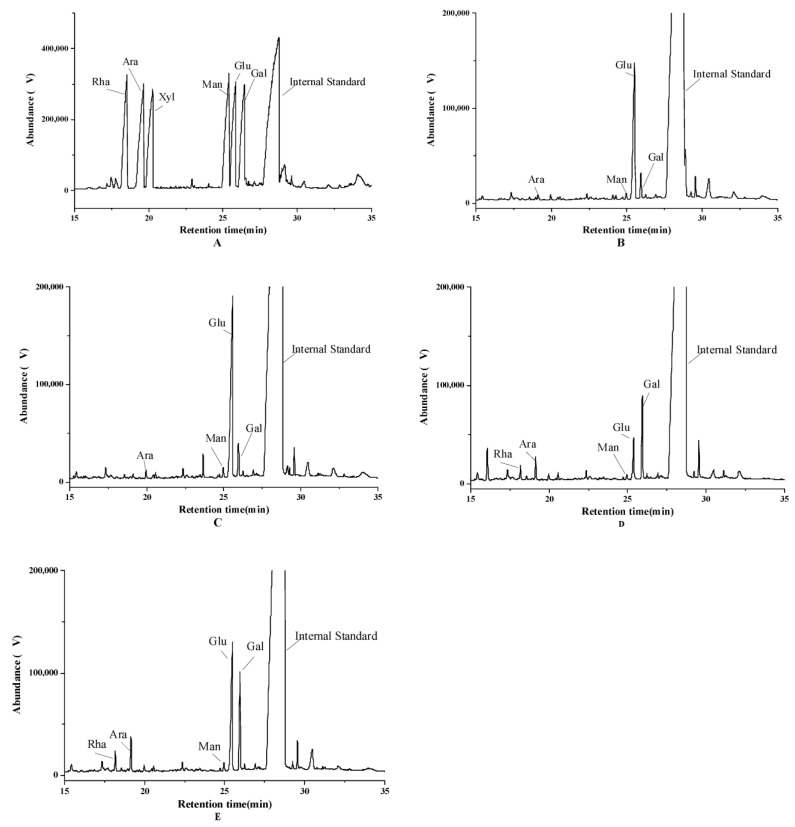
GC-MS chromatography of derivatives of mixture standard monosaccharides. (**A**) mixture standard monosaccharides, (**B**) HD-TG1, (**C**) HD-TG2, (**D**) −20 °C-FT2-HD-TG1, and (**E**): −20 °C-FT2-HD-TG2.

Polysaccharides play important roles in energy storage, structural support and cell wall formation [35]. Monosaccharides can affect the solubility, rheology, stability and biological activity of polysaccharides by influencing their structural characteristics. Figure 5 shows the monosaccharide composition of TG1 and TG2. By comparison with the GC-MS chromatogram of the standard monosaccharide mixture, it was observed that TG1 consisted of arabinose, mannose, glucose and galactose, with molar ratios of 1.0:1.0:1.7:1.8 and 1.0:3.1:100.0:6.7 at HD and −20 °C-FT2-HD, respectively. Meanwhile, TG2 consists of five monosaccharides, rhamnose, arabinose, mannose, glucose and galactose, with molar ratios of 3.3: 5.7: 1.0: 10.9: 20.8 and 2.1: 3.7: 1.0: 22.4: 15.9, respectively. Both TG1 and TG2 are heteropolysaccharides. HD-TG1 −20 °C-FT2-HD-TG1 and −20 °C-FT2-HD-TG2 possessed a large amount of glucose, whereas HD-TG2 had a higher proportion of galactose. It has been shown that the proportion of monosaccharide composition is affected by FT pretreatment. The FT pretreatment did not change the composition of the monosaccharides. However, the molar ratio was changed, which suggests that different pretreatment method conditions lead to a conformational change in the polysaccharide molecular chain [35]. In addition, the content of each monosaccharide increased after pretreatment compared with HD. This may be caused by the fact that the drying time required for FT pretreated samples is shorter than it is for unpretreated samples. This avoids excessive degradation of the polysaccharide components due to long drying time [14]. Feng et al. [14] found that the polysaccharide content of FT1 and FT2 pretreated dried garlic was significantly higher than that of unpretreated dried samples. Xu et al. [15] found that FT pretreatment could not inhibit the enzyme activity and may also aggravate the debranching phenomenon.

#### 3.2.3. Molecular Weight Distributions Analysis

**Figure 6:**
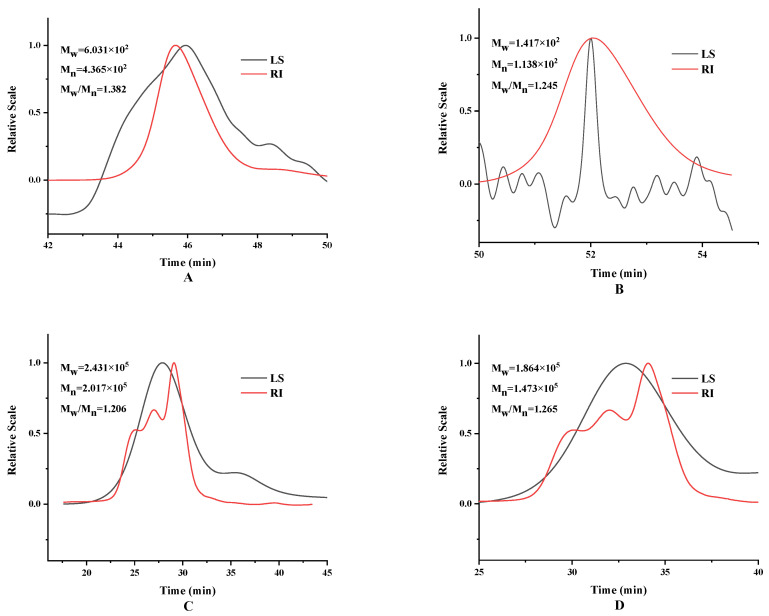
HPSEC-MALLS-RI chromatograms of TG1 and TG2. (**A**) HD-TG1, (**B**) HD-TG2, (**C**) −20 °C-FT2-HD-TG1, and (**D**) −20 °C-FT2-HD-TG2.

The study found that polysaccharides with lower molecular weight have higher antioxidant capacity and enzyme inhibitory activity [11]. The Molecular weight distributions of TG1 and TG2 were determined using the HPSEC-MALL-RT method, and the results are shown in Figure 6. The Molecular weights of HD-TG1 and −20 °C-FT2-HD-TG1 were 6.03 × 10^2^ Da and 1.417 × 10^2^ Da, respectively; the molecular weights of HD-TG2 and −20 °C-FT2-HD-TG2 were 2.43 × 10^5^ Da and 1.864 × 10^5^ Da, respectively. The mass average molar mass (Mw) of polysaccharides obtained from dried samples after FT pretreatment was lower than that of untreated dried samples. The decrease in Mw of polysaccharides could be attributed to the decomposition of polymer clusters or cleavage of the polymer backbone by FT pretreatment [11]. The Mw span of polysaccharides after FT pretreatment was greater than that after HD. This also verifies that FT pretreatment leads to polysaccharide breakdown or cleavage of the main chain. The antioxidant capacity of polysaccharides of relatively low molecular weight is higher, because compared with high molecular weight polysaccharides, the low molecular weight polysaccharides have more reductive -OH terminals for use in the series of reactions with free radicals reactions [14]. Feng et al. [14] found that the polysaccharide Mw of FT pretreated dried garlic was significantly lower than that of control dried samples. It was noted that the Mw of polysaccharides was inversely related to FT cycles. In addition, the degradation of sugars from macromolecules to lower molecules contributes to the accumulation of monosaccharides [36].

### 3.3. Biological Activities of TG1 and TG2

#### 3.3.1. Chemical Antioxidant Activity of TG1 and TG2

**Figure 7:**
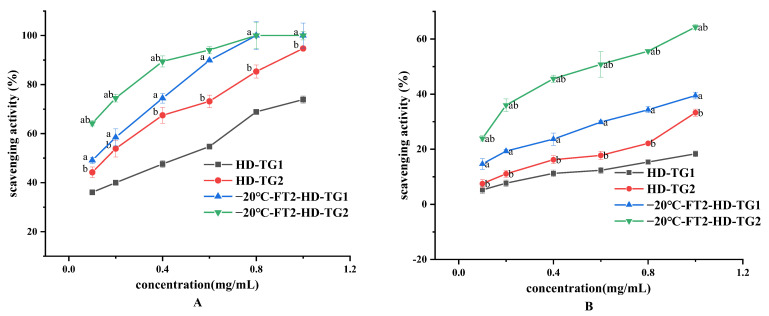
In vitro antioxidant activities of TG1 and TG2. (**A**) scavenging capacity on ABTS+, (**B**) DPPH, ^a^ *p* < 0.05, compared with the same extract at the same concentration of HD; ^b^ *p* < 0.05, compared with the same drying method at the same concentration of TG1.

Effective antioxidant protection is one of the greatest challenges facing food and nutrition sciences. DPPH· and ABTS+ are commonly applied to assess antioxidant capacity of foods. As observed in Figure 7, The free radical scavenging ability of DPPH and ABTS increased with increasing sample concentration. The antioxidant capacity of −20 °C-FT-HD was significantly higher than that of HD extracts (*p* < 0.05). This may be due to the lower molecular weight of −20 °C-FT-HD. The short drying time leads to high retention of active ingredients. At the same concentration, TG2 had higher antioxidant capacity than TG1. According to Mohammad Noshad et al. [29], FT pretreatment improved antioxidant capacity in HD of grapes. This is due to the effect of FT pretreatment on shortening the drying time on enzymatic browning and non-enzymatic oxidative reactions. The antioxidant activity of okra with FT + HD was greater than that with HD, which suggests that the pretreatment combination method influences the antioxidant properties of fruits and vegetables [31].

#### 3.3.2. Cellular Antioxidant Activities of TG1 and TG2

##### Effect of TG1 and TG2 on LDH Levels

**Figure 8:**
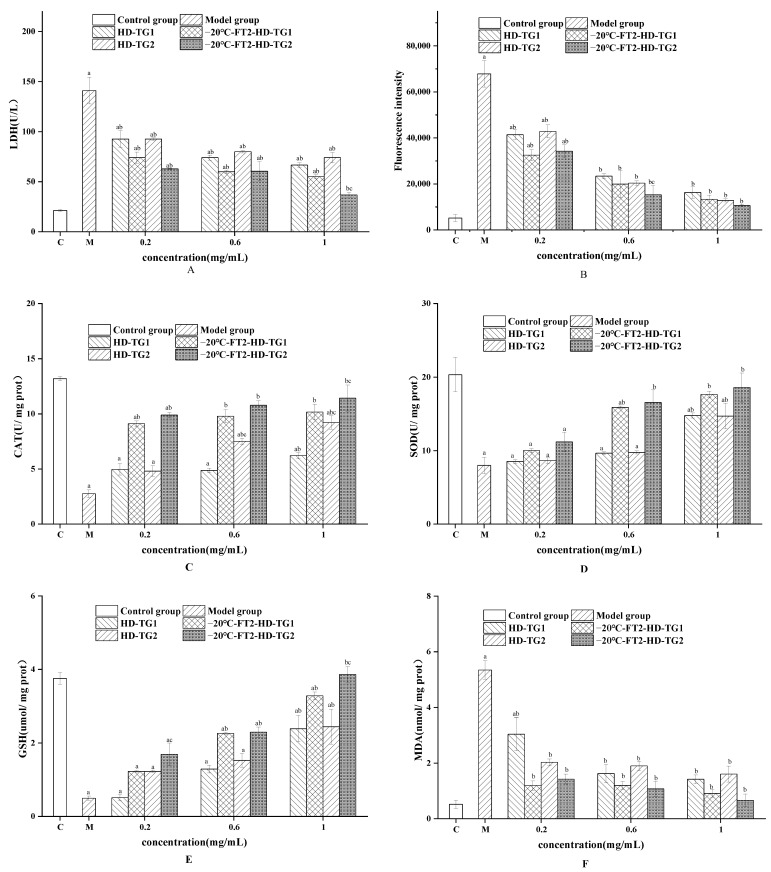
The protective effects of TG1 and TG2 on H_2_O_2_-induced Hep G2 cells, (**A**) LDH, (**B**) ROS, (**C**) SOD, (**D**) CAT, (**E**) GSH, (**F**) MDA, ^a^ *p* < 0.05, vs. control group; ^b^ *p* < 0.05, vs model group; ^c^ *p* < 0.05, TG2 vs. the same drying method and concentration of TG1.

LDH is a cytoplasmic enzyme found in many different cell types and is a recognized indicator of cytotoxicity. Cell membrane damage results in the release of LDH into the surrounding cell culture medium, so the level of LDH in the medium can be used to evaluate whether or not cell membrane damage has occurred. Figure 8A shows the effect of snake gourd extract on LDH levels. Compared with the blank control group, the LDH level in the culture medium increased significantly after H_2_O_2_ treatment (*p* < 0.05), suggesting that H_2_O_2_ treatment induced oxidative damage to the cells. Pretreatment with snake gourd extracts significantly decreased LDH levels in a dose-dependent manner (*p* < 0.05). The LDH levels of −20 °C-FT2-HD extracts were lower than those of the corresponding HD extracts, and lower levels of LDH were released from TG2-treated Hep G2 cells compared with TG1 (*p* < 0.05). Liu et al. [37] reported that rhubarb polysaccharide intervention reduced cellular damage from H_2_O_2_ and reduced LDH leakage and apoptosis.

##### Effect of TG1and TG2 on ROS Levels

Large amounts of ROS can upset the balance between oxidative and antioxidant defenses, leading to cell damage and death. As shown in Figure 8B, the fluorescence intensity of the H_2_O_2_-activated group was significantly greater than that of the control group (*p* < 0.05), suggesting H_2_O_2_ treatment direct injured the biomolecules (lipids, proteins and DNA), even leading to cell damage. The decrease in cellular fluorescence intensity after TG1 and TG2 pretreatment indicated that TG1 and TG2 could alleviate the oxidative damage induced by H_2_O_2_. The fluorescence intensity of −20 °C-FT2-HD was lower than that of the corresponding HD extract, suggesting that −20 °C-FT2-HD was more effective in ameliorating H_2_O_2_-damaged Hep G2 cells. The fluorescence intensity of TG1 (0.6 and 1 mg/mL) was significantly higher than that of the corresponding TG2 (*p* < 0.05). In the report by Sheng et al. [38], selenium polysaccharide from *Platycodon grandiflorum* rescued PC12 cell death induced by H_2_O_2_.

##### Effect of TG1 and TG2 on SOD, CAT, GSH and MDA Levels

SOD, GSH and CAT are effective antioxidant defenses against oxidative stress-induced cell and tissue damage and play an important role in maintaining cellular redox balance [26]. As shown in Figure 8C–E, SOD, GSH and CAT levels were significantly decreased (*p* < 0.05) in the model group compared with the control group, suggesting that H_2_O_2_ induced oxidative stress. On the contrary, intracellular levels of SOD, GSH and CAT increased dose-dependently after pretreatment with TG1 and TG2, suggesting that TG1 and TG2 were beneficial to the amelioration of H_2_O_2_-induced oxidative damage in cells. The −20 °C-FT2-HD extract showed a higher increase in SOD, GSH and CAT activities compared with the corresponding extracts at HD. TG2 showed significantly higher levels of SOD, GSH and CAT compared with the corresponding TG1. Research has shown that many bioactive ingredients have the strong antioxidant capacity to effectively scavenge free radicals and have the ability to stop the oxidative deterioration of food and pharmaceuticals and counteract oxidative stress-induced disease processes in the body. Centaureidin-3-glucoside interfered with the dose-dependent increase of H_2_O_2_-induced activity of SOD, GSH and CAT, protecting cells from oxidative damage [39]. MDA is a major secondary metabolite of lipid peroxidation and can reflect the degree of lipid oxidation. As shown in Figure 8F, H_2_O_2_ significantly increased MDA levels compared with control (*p* < 0.05), indicating cellular oxidative damage. Cellular levels of MDA were dose-dependently reduced after TG1 and TG2 interventions, suggesting that the snake gourd skin extract contributed to the amelioration of H_2_O_2_-induced oxidative damage. Levels of MDA were lower in TG2 compared with the corresponding TG1. Maca root polysaccharide MP treatment could significantly enhance GSH-Px and reduce the levels of LDH and MDA at a dose of 100 mg/kg [26].

#### 3.3.3. Inhibitory Activity against α-Amylase and α-Glucosidase of TG1 and TG2

**Figure 9:**
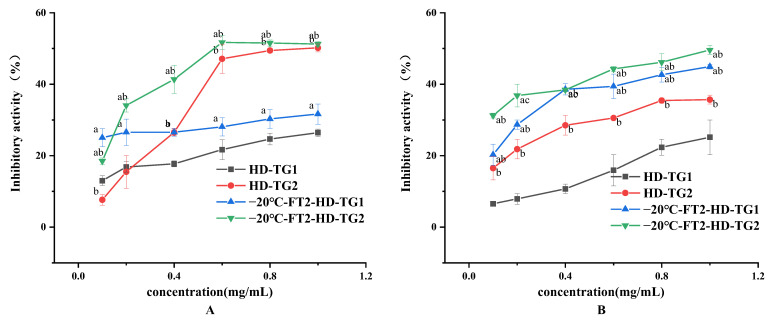
Inhibitory activity against α-amylase and α-glucosidase of TG1 and TG2. (**A**) α-amylase inhibition and (**B**) α-glucosidase enzyme inhibition; ^a^ *p* < 0.05, compared with the same extract at the same concentration of HD; ^b^ *p* < 0.05, compared with the same drying method at the same concentration of TG1.

Control of plasma glucose levels can be facilitated by inhibiting the activity of enzymes involved in the digestion of dietary starch into glucose [40]. Thus, the hypoglycemic properties of TG1 and TG2 can be understood by determining their inhibitory activities against α-glucosidase and α-amylase. As shown in Figure 9, the inhibitory activities of TG1 and TG2 against α-amylase and α-glucosidase increased gradually with the increase in concentration. The −20 °C-FT2-HD samples exhibited higher enzyme inhibition than HD did significantly (*p* < 0.05). TG2 exhibited higher α-amylase inhibition than TG1 did at 0.5–1 mg/mL. As for α-glucosidase, the inhibition rate of TG2 was higher than that of TG1 in all concentration ranges. Compounds including flavonoids, phenolic acids, anthocyanins, saponins, carotenoids, terpenes, sugars, proteins, capsaicinoids, fatty acids and alkaloids have been shown to have the ability to inhibit α-amylase and α-glucosidase activity [40]. The reason for the inhibition of enzyme activity by snake gourd extract may be related to its high polysaccharide and total phenol content. According to Ke et al. [41], vacuum-dried lychee showed a higher performance in α-amylase and α-glucosidase inhibitory activities, attributed to higher content of glyoxylates, proteins and carbohydrates, a higher proportion of effective monosaccharides and lower molecular weight. The −20 °C-FT2-HD-TG2 sample was outstanding in inhibiting α-amylase and α-glucosidase activities, which may be related to the higher polysaccharide content and lower molecular weight.

## 4. Conclusions

In summary, the FT pretreatment had a significant effect on the drying characteristics, active ingredients and biological activities of snake gourd. The mechanism may be that the formation of ice crystals during FT disrupted the internal structure of the snake gourd, leading to the conversion of bound water into free water, and also leading to the rapid transfer of water from the interior to the surface, accelerating the drying rate. The drying process was in accordance with the Page model. FT pretreatment also induced the release of active ingredients as well as the increase of biological activity. Therefore, FT pretreatment is a suitable drying pretreatment technique for producing dehydrated snake gourd. It can reduce the waste of snake gourd resources and improve the utilization rate and economic benefits of snake gourd.

## Figures and Tables

**Table 1:** FT pretreatment methods.

Temperature (°C)	Times			
	0	1	2	3
Unpretreatment	HD	-	-	-
−20	-	−20 °C-FT1-HD	−20 °C-FT2-HD	−20 °C-FT3-HD
−40	-	−40 °C-FT1-HD	-	-
−80	-	−80 °C-FT1-HD	-	-

**Table 2:** Mathematical models of drying.

Model	Equation	References
Lewis	MR = exp(−kt)	[17]
Page	MR = exp(−kt^n^)	[18]
Henderson and Pabis	MR = aexp(−kt)	[19]
Logarithmic	MR = aexp(−kt) + c	[20]
Two-term exponential	MR = aexp(−kt) + bexp(−k_1_t)	[21]
Wang and Singh	MR = 1 + at + bt^2^	[22]

**Table 3:** Results of drying kinetic model fitting of snake gourd with HD and FT pretreatments.

Model	Drying Method	Constants						R^2^	χ^2^	RMSE
		k	k_1_	a	b	c	n			
Lewis	HD	0.34386	/	/	/	/	/	0.95646	0.00521	0.07216
	−20 °C-FT1-HD	0.39438	/	/	/	/	/	0.96845	0.00366	0.06054
	−20 °C-FT2-HD	0.50572	/	/	/	/	/	0.98425	0.00169	0.04113
	−20 °C-FT3-HD	0.48649	/	/	/	/	/	0.98153	0.00205	0.04529
	−40 °C-FT1-HD	0.37694	/	/	/	/	/	0.95654	0.00529	0.07274
	−80 °C-FT1-HD	0.36302	/	/	/	/	/	0.94759	0.00658	0.08112
Henderson and Pabis	HD	0.38065	/	1.12153	/	/	/	0.97059	0.06756	0.06331
	−20 °C-FT1-HD	0.42937	/	1.09775	/	/	/	0.97864	0.00372	0.03722
	−20 °C-FT2-HD	0.52223	/	1.03533	/	/	/	0.98581	0.00266	0.01829
	−20 °C-FT3-HD	0.51058	/	1.05351	/	/	/	0.98505	0.00166	0.01992
	−40 °C-FT1-HD	0.41628	/	1.1183	/	/	/	0.97044	0.00181	0.05759
	−80 °C-FT1-HD	0.40356	/	1.12707	/	/	/	0.96353	0.00384	0.07327
Page	HD	0.17561	/	/	/	/	1.54124	0.99828	2.18 × 10^−4^	0.0037
	−20 °C-FT1-HD	0.2476	/	/	/	/	1.42445	0.99874	1.57 × 10^−4^	0.0022
	−20 °C-FT2-HD	0.41629	/	/	/	/	1.21826	0.99425	6.73 × 10^−4^	0.00741
	−20 °C-FT3-HD	0.37856	/	/	/	/	1.2761	0.99675	3.94 × 10^−4^	0.00433
	−40 °C-FT1-HD	0.20221	/	/	/	/	1.54557	0.99871	1.68 × 10^−4^	0.00252
	−80 °C-FT1-HD	0.17461	/	/	/	/	1.62344	0.99852	1.98 × 10^−4^	0.00298
Logarithmic	HD	0.27146	/	1.23685	/	−0.15933	/	0.98921	0.00145	0.02324
	−20 °C-FT1-HD	0.30942	/	1.21173	/	−0.15385	/	0.99376	0.000836	0.01087
	−20 °C-FT2-HD	0.38083	/	1.14078	/	−0.13992	/	0.99841	0.000205	0.00205
	−20 °C-FT3-HD	0.36588	/	1.17118	/	−0.15384	/	0.99854	0.000194	0.00194
	−40 °C-FT1-HD	0.29421	/	1.24245	/	−0.16693	/	0.98903	0.00153	0.02137
	−80 °C-FT1-HD	0.26773	/	1.28796	/	−0.20953	/	0.98718	0.00184	0.02577
Two-term exponential	HD	0.3805	0.3805	0.56069	0.56068	/	/	0.97059	0.00422	0.06331
	−20 °C-FT1-HD	0.42923	0.42923	0.54878	0.54878	/	/	0.97864	0.0031	0.03722
	−20 °C-FT2-HD	0.52223	0.52223	0.51766	0.51766	/	/	0.98581	0.00203	0.01829
	−20 °C-FT3-HD	0.51056	0.51056	0.52675	0.52675	/	/	0.98505	0.00221	0.01992
	−40 °C-FT1-HD	0.41612	0.41612	0.55915	0.55915	/	/	0.97044	0.00443	0.05759
	−80 °C-FT1-HD	0.40343	0.40343	0.56346	0.56345	/	/	0.96353	0.00564	0.07327
Wang and Singh	HD	/	/	−0.24637	0.015	/	/	0.99409	7.48 × 10^−4^	0.01271
	−20 °C-FT1-HD	/	/	−0.28456	0.02027	/	/	0.99668	4.14 × 10^−4^	0.00579
	−20 °C-FT2-HD	/	/	−0.36091	0.03283	/	/	0.99744	3.00 × 10^−4^	0.0033
	−20 °C-FT3-HD	/	/	−0.34971	0.03084	/	/	0.99906	1.14 × 10^−4^	0.00125
	−40 °C-FT1-HD	/	/	−0.27126	0.01823	/	/	0.99349	8.46 × 10^−4^	0.01268
	−80 °C-FT1-HD	/	/	−0.26057	0.01666	/	/	0.99108	0.0012	0.01793

**Table 4:** Moisture distribution of snake gourd in T2 relaxation spectra.

	HD	−20 °C-FT1-HD	−20 °C-FT2-HD	−20 °C-FT3-HD	−40 °C-FT1-HD	−80 °C-FT1-HD
Bound water (%)	3.06 ± 0.02	2.56 ± 0.08 ^a^	1.87 ± 0.89 ^a^	2.71 ± 0.49 ^a^	2.59 ± 0.49 ^a^	2.26 ± 0.26 ^a^
Immobile water (%)	14.53 ± 0.41	10.99 ± 0.46 ^a^	7.91 ± 4.43 ^a^	10.37 ± 0.06 ^a^	11.67 ± 2.56 ^a^	9.84 ± 0.48 ^a^
Free water (%)	81.77 ± 0.38	86.44 ± 0.51 ^a^	90.21 ± 5.30 ^a^	86.91 ± 0.45 ^a^	85.74 ± 3.05 ^a^	87.89 ± 0.74 ^a^

Each value is expressed as mean ± standard deviation (n = 3). ^a^ *p* < 0.05, vs. HD.

**Table 5:** Rehydration ratio, shrinkage rate and surface color of dried snake gourd slice.

Treatment	Rehydration Ratio (%)	Shrinkage Rate (%)	Surface Color			
			L*	a*	b*	ΔE
HD	7.25 ± 0.17	86.67 ± 0.22	62.88 ± 0.02	1.86 ± 0.04	19.00 ± 0.12	
−20 °C-FT1-HD	4.67 ± 0.17 ^a^	87.73 ± 0.14 ^a^	61.14 ± 0.00 ^a^	0.32 ± 0.04 ^a^	17.37 ± 0.08 ^a^	2.84 ± 0.03
−20 °C-FT2-HD	4.20 ± 0.12 ^a^	88.79 ± 0.11 ^a^	62.24 ± 0.00 ^a^	0.17 ± 0.02 ^a^	16.84 ± 0.03 ^a^	2.82 ± 0.03
−20 °C-FT3-HD	3.34 ± 0.10 ^a^	88.51 ± 0.41 ^a^	64.98 ± 0.01 ^a^	0.23 ± 0.01 ^a^	17.99 ± 0.03 ^a^	2.84 ± 0.02
−40 °C-FT1-HD	4.75 ± 0.13 ^a^	87.46 ± 0.32 ^a^	64.71 ± 0.01 ^a^	−1.93 ± 0.02 ^a^	18.46 ± 0.04 ^a^	4.25 ± 0.01 ^a^
−80 °C-FT1-HD	5.02 ± 0.08 ^a^	87.32 ± 0.49 ^a^	65.27 ± 0.01 ^a^	0.42 ± 0.04 ^a^	19.00 ± 0.08	2.78 ± 0.02

Each value is expressed as mean ± standard deviation (n = 3). ^a^ *p* < 0.05, vs HD. L*, darkness to lightness spectrum; a*, redness to greenness spectrum; b*, yellowness to blueness spectrum; ΔE, total color change.

**Table 6:** Polysaccharide and total phenol content of TG1 and TG2.

Active Compositions	HD-TG1(%)	HD-TG2(%)	−20 °C-FT2-HD-TG1 (%)	−20 °C-FT2-HD-TG2 (%)
Polysaccharide	17.52 ± 0.61	37.93 ± 0.42 ^b^	21.70 ± 0.24 ^ab^	44.34 ± 0.86 ^ab^
Total phenol	1.36 ± 0.06	0.66 ± 0.03 ^b^	1.81 ± 0.09 ^ab^	0.88 ± 0.04 ^ab^

Each value is expressed as mean ± standard deviation (n = 3). ^a^ *p* < 0.05, compared with the same extract at the same concentration of HD; ^b^ *p* < 0.05, compared with the same drying method at the same concentration of TG1. HD-TG1, hot air drying of snake gourd alcohol extract; HD-TG2, hot air drying of snake gourd water extract; −20 °C-FT2-HD-TG1, −20 °C twice frozen–thawed snake gourd ethanol extract; −20 °C-FT2-HD-TG2, −20 °C twice frozen–thawed snake gourd water extract.

## Data Availability

The original contributions presented in the study are included in the article/Supplementary Materials, further inquiries can be directed to the corresponding authors.

## Supplementary Materials

The following supporting information can be downloaded at: https://www.mdpi.com/article/10.3390/foods13131961/s1, Figure S1. Rehydration ratio curve of snake gourd with HD and FT pretreatments.
