# Supplementary figures and images for: Effects of Freeze–Thaw Pretreatment Combined with Hot Air on Snake Gourd (Trichosanthes anguina L.)
Source: Foods. 2024 Jun 21;13(13):1961. doi: 10.3390/foods13131961 (PMC11241399; doi:10.3390/foods13131961)

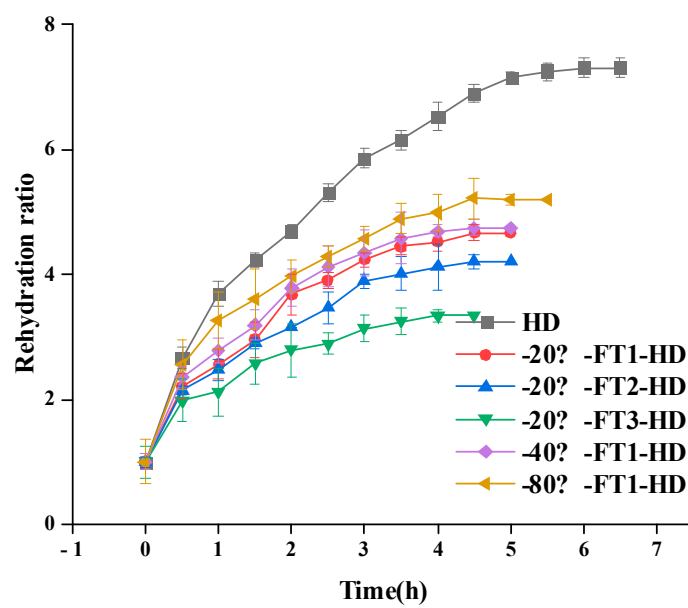

Figure S1 Rehydration ratio curve of snake gourd with HD and FT pretreatments

Supplement: Supplementary file 1 [file foods-13-01961-s001.zip › foods-3063338-supplementary.pdf]
